# Nanoscale Magnetic Effects in CrGeTe_3_—A Review

**DOI:** 10.1007/s10948-025-06987-8

**Published:** 2025-06-28

**Authors:** Avia Noah, Oded Millo, Yonathan Anahory

**Affiliations:** 1 Faculty of Engineering, Ruppin Academic Center, 40250 Emek-Hefer, Monash Israel; 2 The Racah Institute of Physics, The Hebrew University, 9190401 Jerusalem, Israel

**Keywords:** CrGeTe3, Magnetism, Van der Waals materials, Skirmion, Magnetic imaging

## Abstract

Van der Waals (vdW) ferromagnets have garnered extensive attention thanks to their layered structures and the possibility of thinning them down to just a few atomic layers. This review discusses the emergent nanoscale magnetism in CrGeTe₃ (CGT), a 2-D vdW ferromagnet, focusing on its nanoscale properties and potential spintronic applications. We report on local magnetic probe techniques showing that thin CGT films exhibit spontaneous global magnetization at zero field, while thicker flakes display a hard ferromagnetic response only at their edges. We then focus on magnetic edge states in CGT thin films and their potential applications, where direct amorphization of CGT results in magnetic edges similar to those in cleaved films. By fabricating nanopatterned magnetic arrays, it has been demonstrated that tunable magnetic states emerge with anomalous coercivity. Moreover, we present the potential to realize artificial spin-ice configurations through antiferromagnetic dipolar coupling. The review delves into CGT heterostructures, which have demonstrated an anomalous Hall effect, expanding the scope of phenomena accessible in thin magnets. Finally, we discuss observation of magnetic bubbles and skyrmions, which offer additional opportunities for exploring chiral domain structures. The studies of CGT underscore the promise of fundamental investigations into 2-D magnetism while opening new pathways for spintronic applications based on nanoscale magnetic effects and frustration phenomena.

## Introduction

The discovery of two-dimensional (2-D) van der Waals (vdW) materials with long-range magnetic order has opened a new frontier in the field of magnetic materials [1–4]. These materials, characterized by their layered structure, allow for the exfoliation of bulk materials into atomically-thin 2-D crystals, offering an unprecedented range of well-controlled material thicknesses from the bulk to few and even one layers. The properties of 2-D magnetic films often differ from those of their bulk counterparts, and are thickness-dependent, thereby affording unprecedented control over their magnetism [5–8]. This ability to explore magnetism in confined structures has spurred investigations into unconventional magnetic phenomena, unobservable in bulk three-dimensional materials [2–4, 9–11]. A fundamental issue in understanding the magnetic properties of vdW materials is the role of anisotropy that arises mainly from distinct inter-layer and intra-layer exchange interactions [9]. Exfoliating bulk vdW ferromagnets, either conducting such as Fe_3_GeTe_2_ (FGT) [12] or semiconducting such as CrGeTe_3_ [9] (CGT), CrSeTe_3_ [13], CrSBr [14], and CrI_3_ [15], have revealed that ferromagnetism can survive down to the single-layer, where the Mermin-Wagner theorem asserts long-range ordering should be suppressed by thermal fluctuations in the absence of magnetic anisotropy [16].

CGT exhibits a magnetic anisotropy with an out-of-plane (OOP) easy axis, high electron mobility, optical transparency, and strong spin–orbit coupling. Ferromagnetism in CGT was mainly characterized using anomalous Hall effect (AHE) measurements [17, 18], superconducting quantum interference device (SQUID) magnetometry [19], low temperature magnetic force microscopy [20, 21] (MFM), Kerr rotation, SQUID-on-tip [22–24] (SOT), SQUID-on-leaver [25] (SOL), and cryo-Lorentz transmission electron microscopy [26] (CLTEM). Local probe methods, such as SOT, SOL and MFM, are highly effective for investigating magnetization in vdW materials. For example, it was found that thin CGT films exhibit a net magnetization at zero applied magnetic field, a property that diminishes with increasing thickness, where the interior regions of thicker flakes show no net magnetization at zero field, but hard ferromagnetism emerges at the sample edges [22, 23]. The transition from soft to hard ferromagnetism with increasing thickness has also been observed in related vdW materials like Fe_3_GeTe_2_ [27] and CrSiTe_3_ [13], demonstrating the fundamental impact of spatial confinement on magnetic order.

This review focuses on the nanoscale effects in CGT, emphasizing magnetic edges and related phenomena. The review begins by describing the CGT bulk properties, including its crystal structure, electrical, and magnetic properties. It then presents magnetic imaging of CGT, from bulk to the 2-D limit, using techniques such as MFM, SOT, and SOL. The review then delves into cleaved and fabricated magnetic edges exhibiting hard ferromagnetic properties. The capability of directly writing magnetic nanowires and transforming them into hard magnets raises intriguing possibilities for investigating edge effects down to the zero-dimensional limit. Furthermore, the dipolar interactions between such zero-dimensional magnetic dots can result in antiferromagnetic interactions between the nanoparticles revealing its potential in achieving artificial spin-ice. Previous studies have reported AHE in CGT bilayers. Here, we examine those findings and discuss their potential connection to magnetic edges. Finally, we report on the study of magnetic bubbles and skyrmions in CGT thin flakes.

## Magnetic Properties of Bulk CrGeTe_3_

**Fig. 1:**
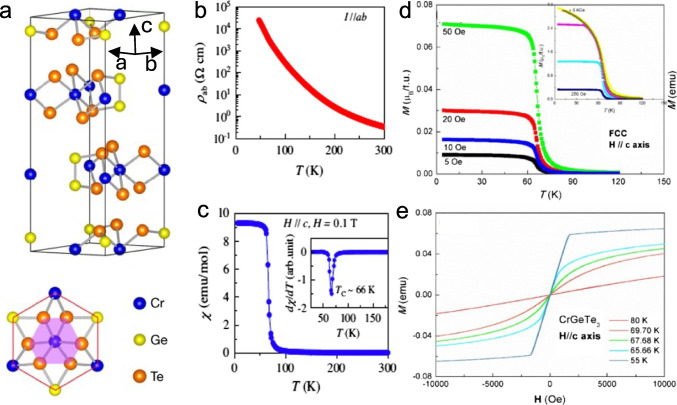
CrGeTe_3_ (CGT) properties. **a** Crystal structure (side and top views) of CGT. Bulk CGT has a layered structure with interlayer vdW coupling (Cheng et al. [9]). **b** In-plane resistivity, ${\rho }_{ab}$, as a function of temperature. **c** Zero field-cooled (ZFC) magnetic susceptibility $\chi$ at $H=0.1$ T applied along the *c*-axis. Inset: temperature derivative of $\frac{d\chi }{dT}$. (Dilip et al. [29]). **d**, **e** SQUID measurements on bulk CGT of the magnetization dependence on temperature M–T for different magnetic fields (data for the highest fields shown in the inset) (**d**) and magnetic field M–H for different temperatures (Tengfei et al. [21])

Figure 1a illustrates the CGT crystal structure, which is defined by a centrosymmetric R $\overline{3 }$ (148) space group. The Te atoms are hexagonally packed in an ABAB stacking sequence along the $c$-axis, with each AB layer forming a network of edge-shared Te octahedra. The Cr atoms occupy octahedral sites in the Te network and are coordinated by six Te atoms. The Cr–Te–Cr bond angle in CGT is approximately 90°, which leads to strong intra-layer superexchange coupling. At ambient pressure, CGT is insulating with a band-gap of approximately 0.7 eV and the in-plane resistivity, ${\rho }_{ab}$, exhibits a typical insulator temperature dependence (Fig. 1b) [28]. The CGT undergoes a Heisenberg-type ferromagnetic transition below ${T}_{c}\approx 66$ K, as shown in Fig. 1c. The bulk magnetic susceptibility, $\chi$, measured with a magnetic field applied along the easy axis ($c$-axis), reveals a sharp transition at ${T}_{c}$. Figure 1d and e presents the results from bulk SQUID magnetometry. The temperature dependence of the magnetization $M\left(T\right)$, shows the expected increase of magnetization at ${T}_{c}$ in presence of an external field along the $c$-axis. The magnetization curve $M(H)$ shows large magnetic response with saturation around 250 mT. However, CGT shows no measurable hysteresis or equivalently no remnant field. This observation challenges the fact that CGT is a ferromagnetic material. The answer may be related to the magnetic microstructure.

## Magnetic Imaging of CrGeTe_3_: From Bulk to Thin-Flakes

**Fig. 2:**
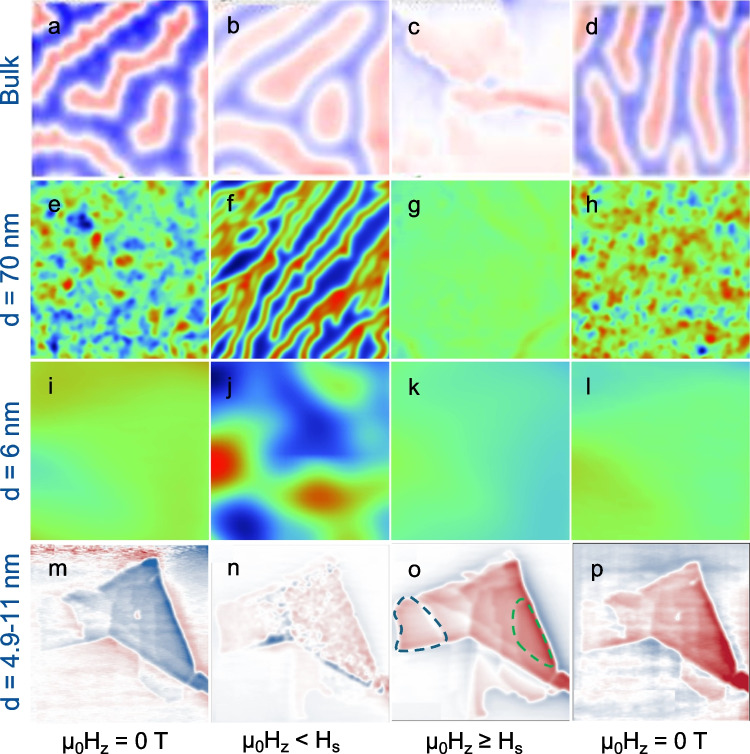
Magnetic imaging of CrGeTe_3_ (CGT) from bulk to the 2-D limit. **a**–**d** MFM images of the out-of-plane magnetic field dependence of magnetic domains. The sequence of images was obtained as it traversed various points of the magnetization vs. magnetic field (Yeonkyu et al. [20]). **e**–**l** Scanning SOT microscopy images ${B}_{z}(x,y)$ of CGT at distinct values of applied out-of-plane field ${\mu }_{0}{H}_{z}$ and sample thickness $d=70$ (**e**–**h**) and $6$ nm (**i**–**l**) (Noah et al. [22]). **m**–**p** Scanning SOL images of CGT flake with 3 to 15 layers (Vervelaki et al. [25]). The blue and green dashed line in o represent layers with *d* = 4.9 and 11 nm (7 and 15 atomic layers), respectively. Image parameters: (**a**–**d**) All MFM images were taken at 4.2 K with tip-sample distance of 400 nm. The scan size is $20\times 20 \mu {m}^{2}$, with $128\times 128$ pixels. ${\mu }_{0}{H}_{z}=$ 0 (**a**), 100 (**b**), 240 (**c**), 0 mT (**d**). **e–l** SOT images (**e**–**h**) $d=$ 70 nm, area scan 5 $\times$ 5 µm^2^, pixel size 40 nm^2^. ${\mu }_{0}{H}_{z}=$ 0 (**e**), 115 (**f**), 175 (**g**), 0 mT (**h**). **i–l** $d=$ 6 nm, area scan 1 × 1 µm^2^, pixel size 30 nm^2^. ${\mu }_{0}{H}_{z}=$ 0 (**i**), 20 (**j**), 120 (**k**), 0 mT (**l**). The blue to red color scale represents lower and higher magnetic field, respectively. **m**–**p** SOL images: area scan $25\times 2$ 5 µm^2^. ${\mu }_{0}{H}_{z}=$ 0 (**e**), 8 (**f**), 40 (**g**), 0 mT (**h**). The blue to red color scale represents lower and higher magnetic field, respectively

The magnetic states of bulk and thin films CGT were investigated extensively using magnetic imaging techniques such as MFM [20], scanning SQUID-on-tip microscopy [22] (SOT), and scanning SQUID-on-lever microscopy [25] (SOL). Figure 2a–p presents images acquired by distinct research groups using the techniques listed above. The images show the magnetic microstructures as a function of the applied magnetic field along the $c$-axis between zero and the saturation field, ${H}_{\text{s}}\sim 300$ mT. Prior to acquiring the first image, the sample was exposed to a field excursion above its saturation field $\left|{H}_{\text{exc}}\right|>\left|{H}_{\text{s}}\right|$. Figure 2a–d depicts the MFM images acquired on bulk CGT. Bulk CGT exhibits a domain structure at zero field (Fig. 2a). The red, blue, and white regions represent positive, negative, and zero magnetization, respectively. The white areas specifically indicate domain walls that separate positive and negative Bloch magnetic domains [30]. As the field approaches saturation, the stripe domains expand, as shown in Fig. 2b. Upon reaching magnetic saturation (Fig. 2c), the MFM image reveals the characteristic saturated magnetic state. When the field is reduced from saturation, stripe domains re-emerge at ${\mu }_{0}{H}_{z}= 220$ mT. We define the demagnetization field ${H}_{d}$ as the field at which magnetic domains re-emerge.

We now turn to the thickness dependence of the saturation and demagnetization fields at thin exfoliated flakes. Figure 2e–l depicts SOT images of CGT flakes with thickness $d=70$ nm (e–h) and $d=6$ nm (i–l). For thicknesses of $d\gtrsim$ 10 nm, the CGT retains its bulk properties. For those samples, at ${H}_{d}$ no magnetic memory was observed, namely the same saturation field is measured with prior excursion at ${\mu }_{0}{H}_{z}=-1$ T [22]. However, for $5<d\lesssim$ 10 nm, the sample remains fully magnetized at zero field (Fig. 2i and l), resulting in an open hysteresis loop (finite remnant field). SOL measurements display the same trend. Figure 2m–p show the magnetization reversal evolution of a CGT flake with $d=3$ to $11$ nm (5 to 15 atomic layers). The blue and green dashed lines in Fig. 2o represent regions in the CGT flakes with *d* = 4.9 and 11 nm (7 and 15 atomic layers), respectively. Starting in the saturated state (${{\mu }_{0}H}_{z}<-140$ mT), the applied field is gradually stepped toward zero. At zero applied field (Fig. 2m), the magnetization in the thicker regions ($d>5$ nm) remains almost unchanged. Upon increasing of ${H}_{z}$, magnetic domains nucleate (Fig. 2n), and with increasing the field ${H}_{z}$, these domains spread over the entire flake, resulting in a complete reversal saturation of the magnetization at ${\mu }_{0}{H}_{z}\sim 40$ mT. For thinner flakes ($d<5$ nm) no hysteretic was observed, with saturation field at ${\mu }_{o}{H}_{z}=20$ mT. We note that the results for flakes with $d<5$ nm appear inconsistent with Kerr measurements, where finite hysteresis was found at six layers [9].

**Fig. 3:**
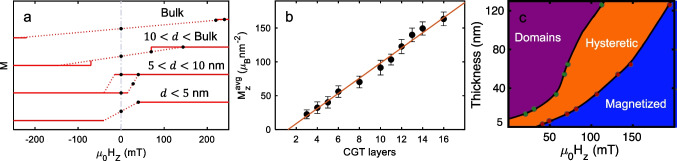
Bulk-to-thin film magnetization. **a** Sketched magnetization curves drawn from ${B}_{z}(x,y)$ measured on film’s parts of different $d$. Dashed lines are a guide to the eye connecting the two saturated fields. The fields at which the images were taken are marked with black dots. **b** ${M}_{z}^{\text{avg}}$ plotted as a function of the CGT layers number and its corresponding linear fit shown with the red line (Vervelaki et al. [25]). **c** A thickness-dependent magnetization-state diagram of CGT shows three states: domains (purple), hysteretic (orange), and magnetized (blue)

For films thicker than 10 nm, the values of ${H}_{s}$ and ${H}_{d}$ are extracted from the magnetic images in Fig. 2, plotted in Fig. 3a, and connected to each other with a dashed line, revealing a bowtie hysteresis loop. Thinner films yield lower values of ${H}_{d}$ and ${H}_{s}$. For $d\lesssim$ 10 nm, the two hysteretic parts of the loop merge and the sample behaves like a conventional hard ferromagnet with an open hysteresis loop (Fig. 3a, $d=6$ nm). Thinner regions of the sample with thickness less than 5 nm, do not show measurable hysteresis and have magnetization curves characteristic of a soft ferromagnet [9]. Transport measurements [22] in CGT/NbSe_2_ and magnetic simulation [25] were performed, supporting the magnetic imaging results. Bow-tie-shaped hysteresis indicates percolating magnetic domains, magnetic vortices, or skyrmion formation during magnetic reversal [31–34]. The magnetic images give a complete picture of the gradual transition of the magnetic hysteresis loop from soft ferromagnetic through hard ferromagnetic to bow-tie shape with increasing thickness, pointing to an evolution of the corresponding magnetic textures with thickness.

Figure 3b shows the averaged reconstructed CGT magnetization ${M}_{z}^{\text{avg}}$, as a function of the number of layers. In particular, the saturation magnetization increases linearly with the number of layers. From the slope of the linear fit, we obtain a magnetization per layer ${M}_{z}^{\text{layer}}=10.9\pm 0.8$ ${\mu }_{\text{B}}$ nm^−2^, equivalent to a saturation magnetization ${M}_{\text{sat}}=2.2\pm 0.2$ ${\mu }_{\text{B}}\text{/Cr}$. In Fig. 3c, we summarize the values of ${H}_{d}$ (green dots) and ${H}_{s}$ (red dots) for different thicknesses. The lines connecting these points constitute boundaries between distinct magnetic states; the domains state (purple), the hysteretic state (orange) and the fully magnetized state (blue). In the domains state, the CGT exhibits small magnetic domains that are insensitive to the excursion field, whereas the opposite holds for the fully magnetized region. In the hysteretic region, the sample can be either in the fully magnetized state or in the domains state depending on the applied magnetic field history.

## Edge Magnetism in CrGeTe_3_

**Fig. 4:**
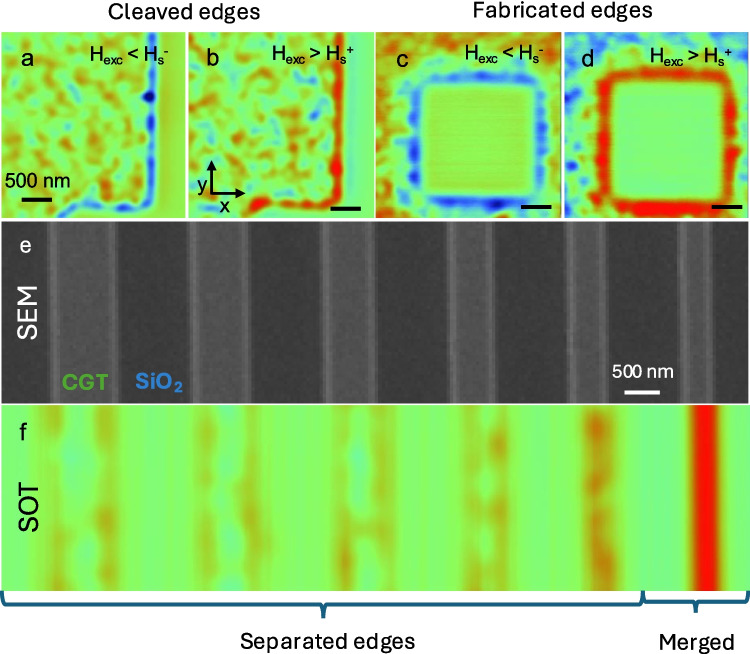
Microscopy images of CrGeTe_3_ edges. **a**, **b** Sequence of natural cleaved CrGeTe_3_ (CGT) flakes acquired at ${\mu }_{0}{H}_{z}=$ 0 mT after distinct field excursions to (**a**) ${H}_{exc}<{H}_{s}^{-}$, (**b**) ${H}_{exc}>{H}_{s}^{+}$. The blue and red edges represent the edge magnetization (Noah et al. [22]). **c**, **d** SOT images of FIB-patterned edges. The image acquired at ${\mu }_{0}{H}_{z}=0$ mT showing magnetic edges in nano-patterned CGT. **e**, **f** From 2-D to 1-D magnetic stripes. CGT patterned into stripes with varying effective widths (${w}_{e}$) and length of 10 µm. **e** SEM image. **f** The SOT image acquired at ${\mu }_{0}{H}_{z}=0$ mT after positive field excursion. For stripes with $w>{w}_{c}$, two distinct magnetized edges (red color scale) separated by a zero average magnetization in the stripe’s interior (color-coded in green). For stripes of width $w<{w}_{c}$ (rightmost stripe), the two edges appear to merge and form a single magnetic domain (Noah et al. [23]). Edge separation width from left to right: $w=770, 660, 550, 460, 400,$ and $270$ nm. Imaging parameters: **a, b** ${\mu }_{0}{H}_{z}=$ 0 mT, area scan 3 $\times$ 3 µm^2^, pixel size 24 nm^2^. The blue to red color scale represents lower and higher magnetic field, respectively, with a shared scale for of ${B}_{z}= 1$ mT. Imaging parameters: **c**, **d** ${\mu }_{0}{H}_{z}=$ 0 mT, area scan $3\times 3$ µm^2^ in size, pixel size 15 nm^2^, acquisition time 5 min/image. **e** ${\mu }_{0}{H}_{z}=$ 0 mT, area scan $2.5\times 10$ µm^2^, pixel size 40 nm^2^, acquisition time 5 min/image. The blue to red color scale represents lower and higher magnetic field, respectively

We now discuss the magnetic properties of the edge and the effect of the edges on the sample’s interior. In Fig. 4a and b, we present two sets of images measured at ${{\mu }_{0}H}_{z}=0$ mT after OOP field excursion to ${{\mu }_{0}H}_{\text{exc}}=-200$ mT $<{H}_{s}^{-}$ (Fig. 4a) and ${\mu }_{0}{H}_{\text{exc}}=200$ mT $>{H}_{s}^{+}$ (Fig. 4b), where ${H}_{s}^{+}$ and ${H}_{s}^{-}$ are the positive and negative saturation fields, respectively. For this sample thickness ($d=24$ nm), magnetic domains appear in the CGT interior as discussed above. However, the edge is clearly magnetized in the same direction as the magnetic field of their respective field excursion. We note that in Fig. 4, negative and positive fields are color coded as blue and red, respectively. We note that hard edge magnetism was observed systematically in all investigated flakes. These observations suggest that the edge is composed of a magnetic nanowire with a width of approximately 10 nm. The magnetic lines presented in Fig. 4a and b are obtained at the edges of a cleaved flake. This raises the question of the emergence of edge magnetism on fabricated edges.

To confirm the capability of fabricating magnetic edges, a square was etched out of a CGT flake using Ga^+^ FIB [23]. Figure 4c–d depicts SOT ${B}_{z}(x,y)$ images the FIB-patterned flake at ${\mu }_{0}{H}_{z}=0$ after a field excursion to ${\mu }_{0}{H}_{z}=-200$ mT $<{H}_{s}^{-}$ (Fig. 4c) and ${\mu }_{0}{H}_{z}=200$ mT $>{H}_{s}^{+}$ (Fig. 4d). In both images, a net magnetic signal measured at the edge of the square highlighting that magnetic edges can be directly written using etching techniques.

The capability to write magnetic edges allows a systematic study of the edge magnetic properties as a function of lateral dimensions. Figure 4e presents a SEM image of 10 µm long CGT stripes etched using Ga^+^ FIB with varying widths ($w$). Figure 4f depicts a ${B}_{z}\left(x,y\right)$ image of the stripes acquired at ${\mu }_{0}{H}_{z}=0$ after a field excursion at ${\mu }_{0}{H}_{z}=200$ mT. The images of the wider stripes feature two distinct magnetized edges (color-coded in red) separated by a zero-average magnetization in the stripe’s interior (color-coded in green). Narrower stripes result in a shorter distance between the edges. For the second to last stripe on the right ($w=400$), the distance between the edges is below the SOT resolution. For the narrowest (rightmost) stripe, of width smaller than a critical width ${w}_{c},$ the ${B}_{z}(x)$ signal is four times larger than the signal at a single edge, suggesting that the presence of magnetic edges renders the sample interior a hard ferromagnet. Further quantitative analysis of the signal demonstrates that indeed the sample interior magnetic properties are determined by the magnetic edges.

**Fig. 5:**
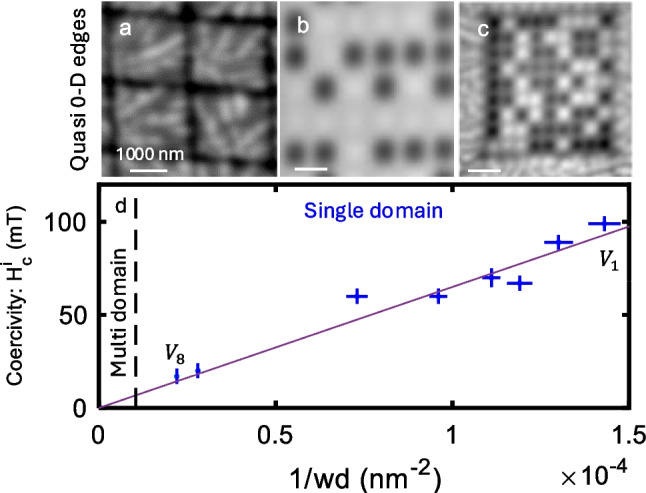
Average coercivity of CrGeTe_3_ (CGT) islands. **a**–**c** Images of island arrays patterned via FIB with island dimensions $d=60$ nm for all arrays, and widths, $w=1600$ nm (**a**), $600$ nm (**b**), and $150$ nm (**c**). The nanoparticle characteristic size $D$ determine the island magnetic state and coercivity. For islands with characteristic size larger than ${D}_{s}$ as in **a**, $w=1600$ nm $>{D}_{s}$, the islands interior breaks to domain, while its edges are magnetized. **b**, **c** For islands with characteristic size smaller than ${D}_{s}$ a single magnetic domain appears due to edge confinement. The images acquired near the coercive field of the corresponding arrays. **d** The median island coercive field, ${H}_{c}^{i}$ ($\widetilde{{H}_{c}^{i}})$, as a function of the parameter $w/V=1/wd$ (Noah et al. [24]). Imaging parameters: **a** ${\mu }_{0}{H}_{z}=$ 70 mT, area scan $4.1\times 4.1$ µm^2^, pixel size 32 nm^2^, **b** ${\mu }_{0}{H}_{z}=$ 20 mT, area scan $11\times 11$ µm^2^, pixel size 115 nm^2^, and (**c**) ${\mu }_{0}{H}_{z}=$ 100 mT, area scan $4.2\times 4.2$ µm^2^, pixel size 30 nm^2^. The scale bar is 1000 nm in all images. The black to white color scale represents lower and higher magnetic fields, respectively

The lateral edge confinement raises the question of the effect of confinement in both $x$-$y$ directions. In particular, how does the perimeter-to-volume ratio affects the magnetic properties of CGT. To answer this question, square-shaped CGT nanoislands with a wide range of sizes and aspect ratios were investigated [24]. Figure 5a–c displays magnetic images of islands acquired near the array’s coercive field, where the black/white color-coded area indicates a magnetic moment pointing downwards/upwards. The array parameters of the islands are: thickness, $d=60$ nm for all, and widths, $w=1600, 600$, and 150 nm respectively. The results indicate that small islands ($w\le 600$ nm, Fig. 5b, c), are single-domain, while larger islands with $w=1600$ nm, exhibit stable multiple magnetic domains (Fig. 5a).

The coercive field of the array ${H}_{c}^{a}$ is reached when $M\left({H}_{c}^{a}\right)=0,$ or equivalently when the magnetization of half of the islands points in a given direction. Therefore, ${H}_{c}^{a}$ is the median value of the field at which a single-island reverses its magnetization, noted as $\widetilde{{H}_{c}^{i}}$. Notably, $\widetilde{{H}_{c}^{i}}$ varies significantly with the island geometry, and ranges from 15 to 100 mT. In the absence of a stable magnetic domain wall, the magnetic saturation field is ${H}_{c}^{i}=2K/{m}_{i}$, where $K$ is the island magnetic anisotropy and ${m}_{i}$ is the magnetic moment of a single island. Neglecting thermal activation effects, ${H}_{c}^{i}$ should be independent of the island size because $K$ and ${m}_{i}$ both scale as the volume, $V$. Finite-temperature effects tend to reduce the particle coercivity for small volumes. In striking contrast, the results reveal an anomalous size-dependent coercivity, with smaller islands exhibiting higher coercivity. Figure 5d presents a surprising linear relation between the measured $\widetilde{{H}_{c}^{i}}$ and $1/wd$. Assuming that ${m}_{i}\propto V$ (as shown in Fig. 3b), a $1/wd$ dependence implies that the magnetic anisotropy depends on the perimeter, i.e. $\propto w$. This experimental finding demonstrates the one-dimensional nature of the magnetic edge state shown in Fig. 4.

**Fig. 6:**
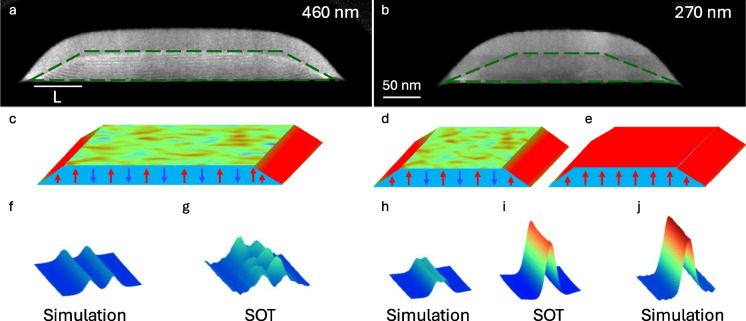
Origin of the magnetic edge in CrGeTe_3_. **a**, **b** Scanning transmission electron microscope (STEM) cross-sectional images measured in the middle of the stripe (Fig. 4e) with effective widths ${w}_{e}=460$ nm (**a**) and ${w}_{e}=270$ nm (**b**). The effective crystalline CrGeTe_3_ (CGT) region is marked with green dashed lines. **c**–**e** Schematic illustration of the local magnetic structure at the edges and interior for different stripe widths. In panels **c** and **d,** the edges but not the interior of the sample retain their magnetization. In panel **e**, the whole stripe is a hard ferromagnet. **f**–**j** A comparison between the SOT images and magnetostatic simulations. **f**, **h** Simulations of the stripe magnetization resulting from the magnetized edges for a triangular cross-section, marked by red in panels **c** and **d**. **j** Simulations of the stripe magnetization assuming that the whole stripe is magnetized, marked by red in panel **e**. **g**, **i** ${B}_{z}(x,y)$ SOT images of the stripes in **a** and **b**. The blue to red color scale represents lower and higher magnetic fields, respectively, with a shared scale of $3$ mT (Noah et al. [23])

Attempting to elucidate the origin of magnetization at the CGT edges, cross-sectional scanning transmission electron microscopy (STEM) images of a FIB-patterned stripe sample were acquired, as seen in Fig. 6a. The geometry of the sample, as obtained from the STEM cross-section, is trapezoid-shaped. Part of the stripe is amorphized during the FIB pattering—residing above the marked green dashed line in each sample. Consequently, the effective part contributing to the magnetization is marked by this dashed line, also having a trapezoidal geometry. Given this criterion, the effective stripe width is defined as ${w}_{e}=\frac{{w}_{\text{base}}+{w}_{\text{top}}}{2}$, as determined from the dashed line, yielding ${w}_{e}=$ 460 nm (Fig. 6a) and 270 nm (Fig. 6b) with an effective thickness of ${d}_{e}=50$ nm. Importantly, the edge of the sample has a tapered cross-section. The STEM images reveal a variation of the flake structure on the edge, where its thickness is substantially diminished. Due to the reduced dimensionality of the edge, it is reasonable to postulate that the thinner edge behaves as the thin CGT flake (< 10 nm), thereby exhibiting finite magnetization at zero field.

Magnetostatic simulations were carried out to understand this observation quantitatively. For a wider stripe (${w}_{e}=460$ nm, Fig. 6a), the edge model by assuming a right-angled triangle cross-section with area $L\times d/2 =2000$ nm^2^ (Fig. 6a). The simulated field distribution emanating from such a triangular cross-section edge (Fig. 6f) is in good agreement with the measured SOT image (Fig. 6g). Thus, the simulation confirms a magnetic edge width of a few tens of nanometers. Figure 6d presents the same calculation executed for the narrow stripe (${w}_{e}=270$ nm), by modeling the edges as a triangle cross-section of $L\times d/2=2500$ nm^2^. In contrast to the wider stripe, this yields a poor agreement between the simulation (Fig. 6h) and the SOT results (Fig. 6i), where the simulated signal magnitude is smaller than the experimental data by a factor of four. To obtain good agreement, one needs to assume that the entire stripe is magnetized as illustrated in Fig. 6e and simulated in Fig. 6j. Therefore, the edges enhance the remanent magnetization in the sample interior, resulting in a proximity-induced hard ferromagnetic state. The typical decay length of such interaction can be estimated as about ${w}_{c}=300$ nm for ${d}_{e}=50$ nm and this defines the critical width ${w}_{c}$ for the emergence of hard FM in the sample interior.

## Possible Microscopic Mechanism for Edge Magnetism

**Fig. 7:**
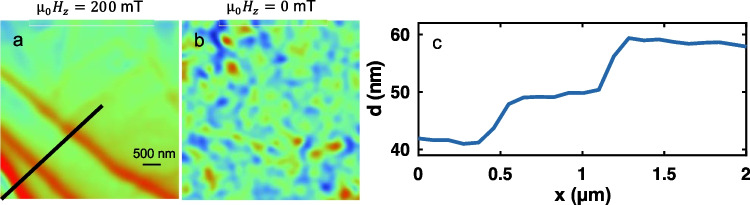
SOT image of CrGeTe3 terraces with a corresponding atomic force microscopy line scan. **a**, **b** SOT magnetic image at ${\mu }_{0}{H}_{z}=200$ mT (**a**), and ${\mu }_{0}{H}_{z}=0$ mT (**b**). The magnetic domains evolve with no relation to the step-edges. **c** Atomic force microscopy height profile measured along the black line presented in **a** resolving steps of ∼ 10 nm. Image parameters: area scan 5 × 5 $\mu$m^2^, pixel size 26 nm. The blue to red color scale represents lower and higher magnetic field, respectively, with a scale of ${B}_{z}=1$ mT (Noah et al. [22])

Edge effects are omnipresent in physics and their origins are diverse. The origin of edge magnetism in CGT, however, is still unknown. Several mechanisms were considered in the past [22–24]. One of them is related to the in-plane dangling bonds. Magnetic edges were found at the edges of naturally cleaved samples exposed to air, encapsulated with another material [22] and self-encapsulated with amorphized CGT [23]. The nature of the dangling bond in these three cases should be different and thus the observation of edge magnetism in all these cases suggests that the effect of in-plane dangling bonds is negligible. Moreover, in-plane dangling bonds are also present at step-edges between two terraces. Figure 7a shows SOT magnetic image at ${\mu }_{0}{H}_{z}=200$ mT, here the step edges are visible with the SOT because the total magnetization is function of the thickness (see line scan in Fig. 7c). However, at ${\mu }_{0}{H}_{z}=0$ (Fig. 7b), no magnetic edge is visible [22]. The absence of magnetic edges at step-edges further demonstrates that the in-plane dangling bonds scenario is less probable. This observation shows that the vanishing sample thickness is necessary for the appearance of edge magnetism.

Gallium contamination during the FIB process was also considered as a potential mechanism. Although Ga ion irradiation is known to alter magnetic properties in other related materials [35], previous results indicate that Ga contamination does not account for the observed edge magnetism in crystalline CGT [23] (see Supporting Note 3 and Figs. 4–6 in Noah et al. [23], where magnetic edges were found at the edge of flakes that were never exposed to the FIB Ga beam.) Another plausible mechanism is the presence of strain at the sample edges [36, 37]. It is plausible that strain appears at low temperatures at the interface between the amorphous and crystalline CGT regions. However, given that such magnetic edge state was found in cleaved samples, where such an interface does not exist, tends to rule out this scenario.

Recently, similar edge state in another material is thought to be caused by the Stoner mechanism [38]. Notably, applying this model to CGT is not straightforward since CGT is insulating and further investigation will be necessary to examine the possible presence of Stoner-type magnetism in CGT.

## Field-Induced Antiferromagnetic Correlations in Nanopatterned CGT

The CGT nano-island coercivity scales as $1/wd$, which suggests that the magnetic anisotropy is proportional to the perimeter, further highlighting the important role played by edge magnetization. Another effect that could influence the island properties is the inter-island dipolar interaction. Each island can be considered as a local magnetic dipole pointing out of the plane, generating a dipolar field ${\overrightarrow{B}}_{dip}=-\frac{{\mu }_{0}m}{{r}^{3}}\widehat{c}$ in a neighboring island located at a distance *r* ($\widehat{c}$ is the unit vector normal to the plane). This should result in an effective antiferromagnetic inter-island interaction. However, at ${H}_{z}=0$, all islands remain magnetized in the same direction, showing that the magnetic anisotropy term dominates the dipolar interaction. Assuming an arbitrary precision on the applied magnetic field, the energy barrier can be made arbitrarily small to let the ${B}_{dip}$ term dominate. Randomizing effects such as finite-temperature and inhomogeneous islands can also compete with the effective antiferromagnetic interactions. Assuming that the dipolar interaction is larger than the randomizing effects at ${H}_{c}$, we expect nearest neighbors to point in opposite directions.

**Fig. 8:**
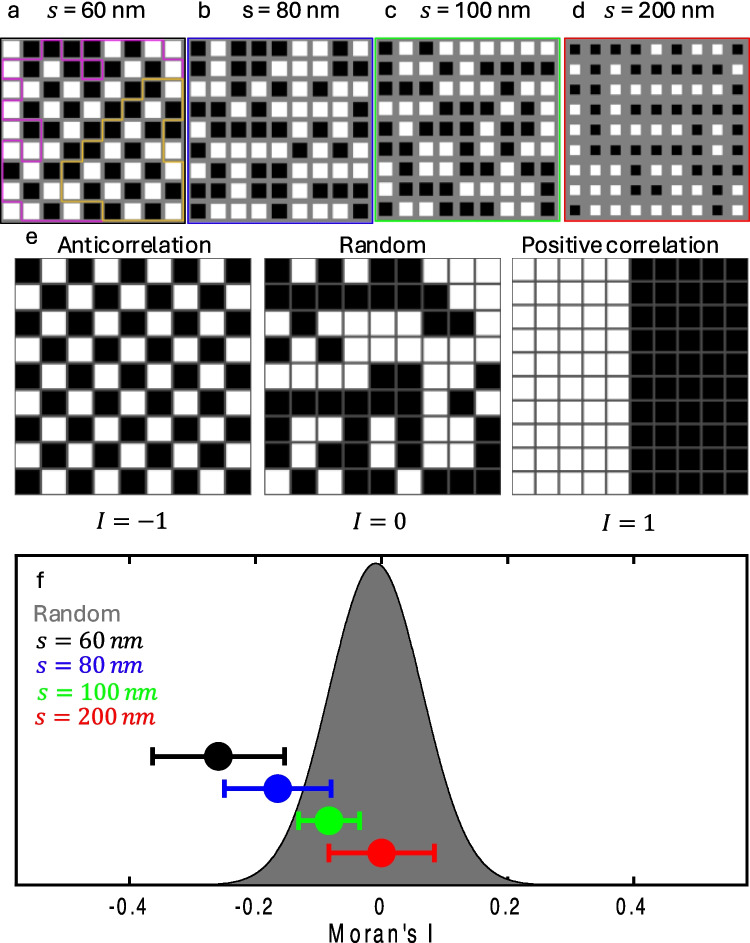
Separation dependent inter-island interactions.** a**–**d** Sequence of binary matrices computed from the SQUID-on-tip (SOT) ${B}_{z}(x,y)$ images for island separations of $\text{s}=60$ (**a**), $80$ (**b**), $100$ (**c**), and $200$ nm (**d**) (islands thickness—*d* = 35 nm). The purple and gold lines delineate two fully anticorrelated sublattices. The black/white color scale represents the magnetic moments pointing down/up, and the gray represents amorphous CGT. The SOT images were acquired at the relevant coercive fields, ${\mu }_{0}{H}_{c}=$ 68 (**a**), 89 (**b**), 99 (**c**), and 70 mT (**d**). **e** Illustration of matrices at the coercive field ($M\left({H}_{z}={H}_{c}\right)=0$) with different types of inter-island correlations: A checkboard matrix that shows perfect anticorrelation with Moran’s $I = -1$ (left). A random matrix, which has zero spatial correlation $I=0$ (center). A perfectly correlated matrix for which $I=1$. **f** Gray: distribution of Moran’s $I$ values calculated for random $9\times 9$ matrices at the coercive field based on a set of 10^4^ trials. Distinct markers represent the average Moran’s $I$ measured for CGT arrays at ${H}_{c}$ for separations of $s=60$ (black), $80$ (blue), $100$ (green), and $200$(red) nm. The markers indicate the mean values, and the error bars the range within one standard deviation. $I=0.00\pm 0.08, -0.08\pm 0.05, -0.16\pm 0.08,\text{ and}-0.25\pm 0.1$ for $s=100, 80$, and $60$ nm, respectively (Noah et al. [39])

To investigate the interaction between the islands, arrays with a thickness $d=35$ nm and four distinct inter-island separations $s=60, 80, 100,$ and $200$ nm were fabricated using the Ga^+^ FIB [39]. The distance between the islands centers was kept constant at 300 nm, resulting in an effective magnetic island width of $w=300-s$ nm (*s* is the width of the separation line). Figure 8a–d depicts the binary representation of the magnetic arrays at the coercive field, ${H}_{c}$, where $M\left({H}_{z}={H}_{c}\right)\sim 0$. The emerging magnetic patterns for $s=60$ nm (Fig. 8a), reveal a propensity for each island to orient the magnetization in a direction antiparallel to its neighbors. Conversely, as observed in Fig. 8d ($s=200$ nm), more distant neighboring islands are more often magnetized in parallel to each other, forming larger domains. To quantify the strength of the antiferromagnetic correlation as a function of the inter-island separation, $s$, a Moran’s $I$ metrics where employed [40, 41]. $I$ is calculated from the following expression:

$$I=\frac{n}{{\sum }_{i}{\sum }_{j}{w}_{i,j}}\frac{{\sum }_{i}{\sum }_{j}{w}_{i,j}\left({m}_{i}-\overline{M }\right)\left({m}_{j}-\overline{M }\right)}{{\sum }_{i}{\left({m}_{i}-\overline{M }\right)}^{2}} ,$$

where $n$ is the number of islands, ${m}_{i}$,${m}_{j}$ refer to the island magnetization in the location $i$, $j$, $\overline{M }=M({H}_{z})/{M}_{\text{Sat}}$ is the normalized mean value of the array magnetization, and ${w}_{i,\,j}$ is the spatial weight between cells. To consider the nearest neighbor correlation, we set ${w}_{i,j}=1$ between nearest neighbors, while all the other of the ${n}^{2}\times {n}^{2}$ matrix entries are set to zero. The $I$ values range between − 1 and 1, with a value of − 1 indicating perfect negative correlation and a dominant antiferromagnetic interaction (Fig. 8e, left panel). $I=0$ indicates the absence of any correlation, as expected for a randomly distributed matrix (Fig. 8e, central panel). A value of 1 is obtained in the case of perfect positive correlation (ferromagnetic interaction), which would result in two magnetic domains pointing in opposite directions (Fig. 8e, right panel).

Figure 8f presents a distribution of Moran’s $I$ values calculated from computer-generated $9\times 9$ random matrices at ${H}_{c}$ ($M({H}_{z}={H}_{c})=0$) and based on a set of 10^4^ trials (gray distribution). This distribution is centered around $I=0$, highlighting zero spatial correlation in the absence of dipolar interactions. Each marker plotted in Fig. 8f represents the experimentally determined mean $I$ values at ${H}_{c},$ obtained from distinct field sweeps. The error bars represent the obtained standard deviation. For the largest separation ($s=200$ nm), an $I=0.00\pm 0.08$ obtained, which indicates negligible interaction (Fig. 8f, red marker). Conversely, significant anticorrelations between the islands are observed for $s<100$ nm ($I=-0.08\pm 0.05, -0.16\pm 0.08, \text{and}-0.25\pm 0.1$, for $s=100, 80,$ and $60$ nm, respectively).

By nanopatterning a 2-D vdW magnet in a square grid one can finely adjust interactions to create states not accessible in isolated pristine flakes, which exhibit zero net magnetization at zero field. Such lab-made structures provide an exciting playground to investigate a wide class of fundamental correlated phenomena. Further investigations could combine the effective antiferromagnetic interactions, mediated by the dipolar field with other lattices that induce geometric frustration to study artificial spin ice in magnetic 2-D materials. These phenomena are not beyond reach thanks to lithography techniques, and the flexibility in sample fabrication using vdW material technology.

## The Anomalous Hall Effect in CGT Bilayer

**Fig. 9:**
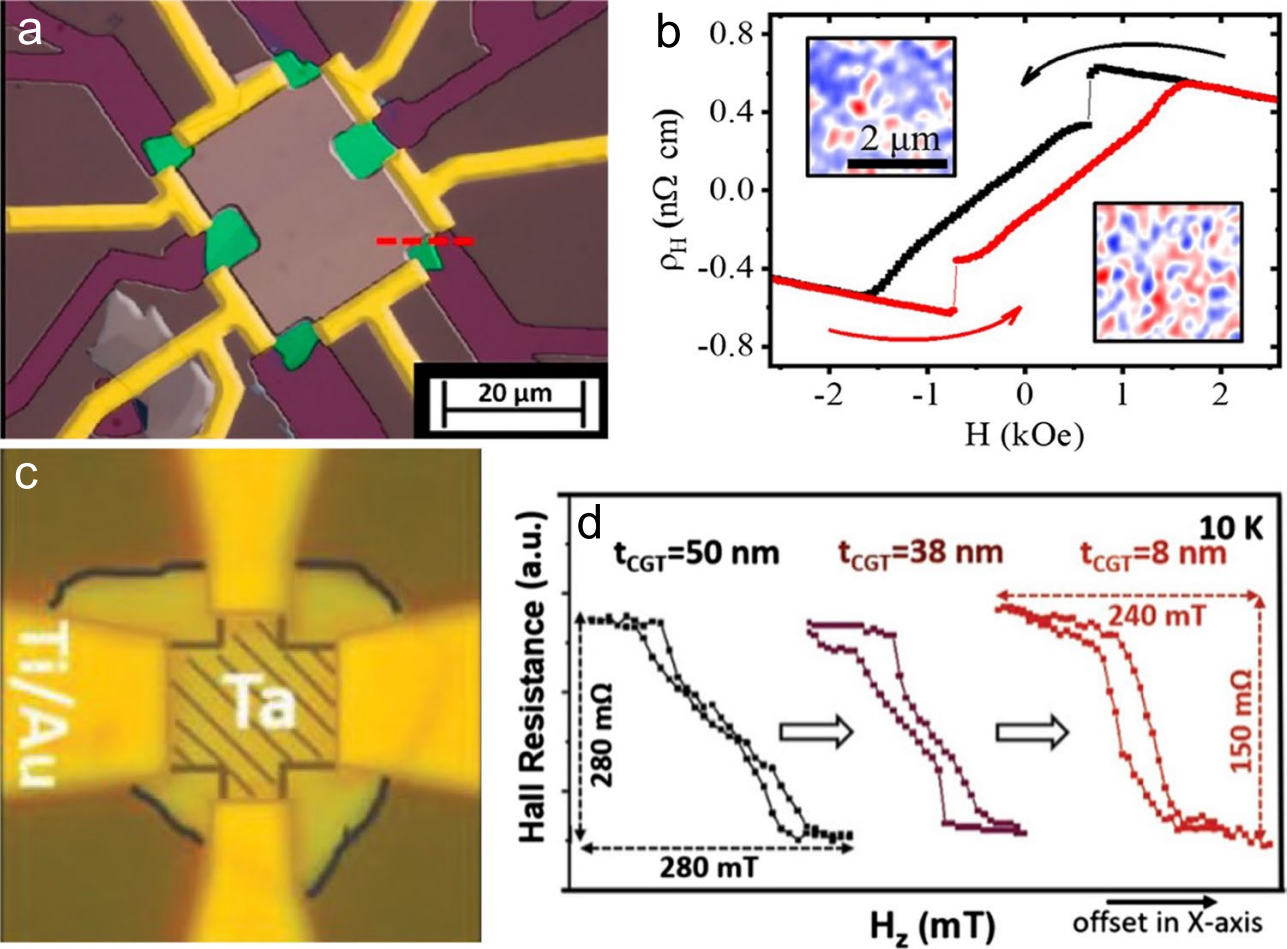
Probing the anomalous Hall effect (AHE) in CrGeTe_3_ (CGT) heterostructures. **a** Optical micrograph of a Pt/CGT heterostructure with false color to clarify different regions of the device. False coloring is used to distinguish the different material layers: Pt (5 nm) in gray, CGT (30 nm) in green, Au in yellow, and SiO_2_ in dark red. **b** Anomalous Hall hysteresis loop in the Pt/CGT bilayer, measured at 4 K. Insets: MFM images of CGT acquired at different magnetic fields within the hysteretic state (Lohmann et al. [18]). **c** Optical image of a fabricated Hall bar device from a Ta/CGT heterostructure and measurement setup. **d** Normalized ${R}_{\text{AHE}}$ for CGT thicknesses of *t*_CGT_ $=50, 38,$ and $8$ nm. All three hysteretic curves exhibit similar AHE behavior (Ostwal et al. [17])

The reported critical thickness below which CGT remains magnetized at zero field is approximately 10 nm. However, several studies showing AHE in bilayers containing CGT were reported, suggesting that CGT can exhibit magnetic memory at zero field, as indicated by finite ${R}_{xy}$ at ${\mu }_{0}{H}_{z}=0$ [17, 18]. Lohmann et al. observed an AHE signal in a CGT/Pt heterostructure, where a 5 nm thick Pt film was deposited on exfoliated CGT flakes with thicknesses between 20 and 50 nm. The AHE hysteresis loops persisted up to approximately 60 K, matching the Curie temperature of CGT. The slanted AHE loops, featuring a narrow opening, indicate magnetic domain formation, which was confirmed by magnetic imaging. Figure 9a shows a representative Pt/CGT device with a $\sim 35$-nm-thick CGT flake. Figure 9b displays the induced AHE data in Pt, measured at 4.2 K with a current of 2.0 mA under an applied OOP magnetic field. We note that the Hall resistivity signal is rather small, less than nΩ-cm centimeter. Given that Pt is paramagnetic, no AHE signal is expected for a Pt film. Therefore, the AHE in the Pt/CGT device, which appears below the CGT ${T}_{c}$, is necessarily induced by the ferromagnetic CGT, and the AHE hysteresis in Pt thus merely reflects the magnetic hysteresis of the underlying CGT.

The magnetic images in Fig. 2 indicate the absence of net magnetization at zero field in the interior for flakes thicker than 10 nm under zero external magnetic field. This non-magnetic behavior is in contrast with the observation of anomalous Hall signals for Pt/CGT bilayers with CGT thicknesses larger than 10 nm depicted in Fig. 9. This apparent contradiction may be attributed to CGT being proximitized to materials with strong spin–orbit coupling, such as Pt or topological insulators (TIs) [28, 42]. Magnetic anisotropy is heavily influenced by the material’s spin–orbit coupling, and proximity effects can modify this property, thereby altering the magnetic characteristics of the ferromagnet interface.

Figure 9c presents an optical image of a Ta/CGT Hall bar device. In Fig. 9d, the normalized AHE curves for CGT thicknesses of 50, 38, and 8 nm are displayed, all exhibiting distinct hysteresis loops. These curves reveal a similar transition across the different thicknesses as observed by magnetic imaging. The emergence of more pronounced hysteresis in thinner samples indicates an enhancement of the perpendicular magnetic anisotropy, which is consistent with the transition from a bow-tie to an open hysteresis loop illustrated in Fig. 3a.

Another explanation for the observation of AHE in bilayers CGT films thicker than 10 nm could involve the existence of magnetized edges in the underlying CGT film, where the AHE signals in the Pt or Ta over-layers result from the edge magnetization. Future research should focus on understanding the origins of the AHE in such heavy-metal/CGT heterostructures. This issue might be resolved by comparing two sets of devices: one where one or more edges of the CGT flake overlap with the Hall bar area and another where no edge overlaps with the Hall bar. Additionally, comparing ${B}_{z}\left(x,y\right)$ magnetic images of regions with and without the overlaying conductor could provide insights into the influence of the conducting layer on the flake’s magnetization. If it turns out that edge magnetization, which represents a vanishingly small fraction of the sample, is responsible for the finite AHE signal, it could not only explain the origin of the AHE but also serve as a sensitive transport probe for edge magnetization in 2-D materials.

## Study of Magnetic Bubbles and Skyrmions in CrGeTe_3_

**Fig. 10:**
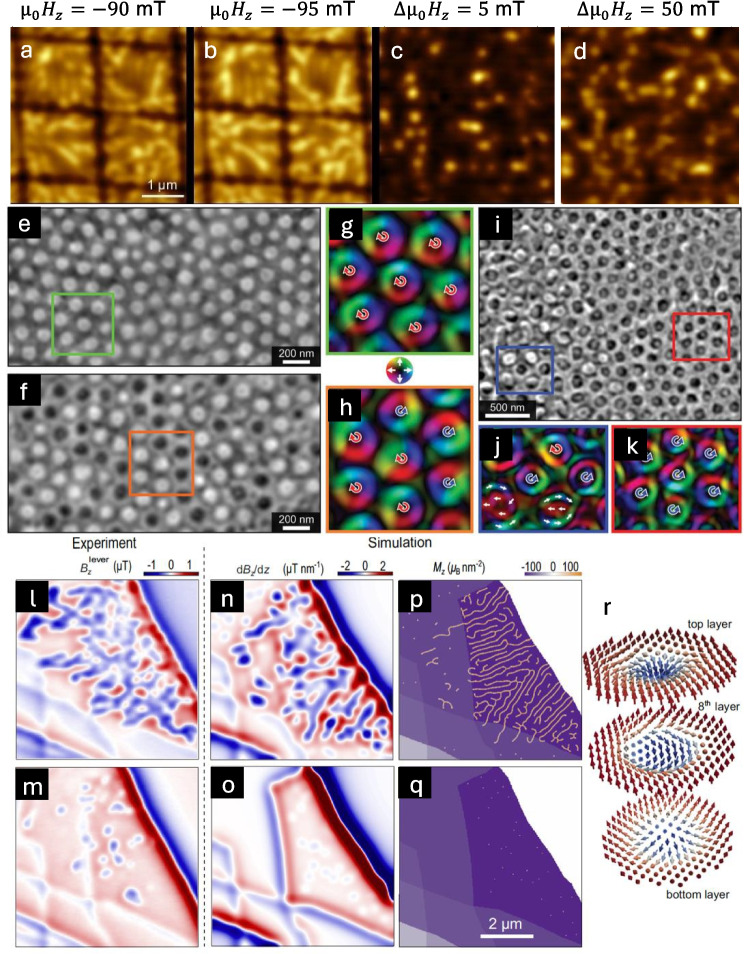
Magnetic bubbles in a CrGeTe_3_ (CGT) thin flakes. **a**–**d** SOT images of magnetic bubbles in CGT. **a**, **b** Subsequent SOT images taken at ${\mu }_{0}{H}_{Z}=-90$ and $- 95$ mT. **c** Subtracting **a**, **b** SOT images with $\Delta {\mu }_{0}{H}_{Z}=5$ mT resolve in magnetic bubble formation. **d** Subtracting two SOT images with $\Delta {\mu }_{0}{H}_{Z}=50$ mT. **e**–**k** Magnetic bubbles of different chirality in CGT. **e**, **f** Cryo-Lorentz transmission electron microscopy, transport of intensity equation (TIE)—reconstructed phase images of the same sample region after two different field-cooling experiments, showing a homochiral bubble lattice in **e** and a mixed-chirality bubble lattice in **f**. Bubbles with right-handed chirality are bright, and bubbles with left-handed chirality are dark. **g**, **h** Integrated magnetic induction maps of the green and orange boxes in (**e**, **f**) (chirality is indicated by red or blue arrow). **i** Phase image of a region of a CGT flake that has irregular topography. The right side of the sample is flat, and the left side is approaching a wrinkle in the flake. **j**, **k** Magnetic induction maps of the curved and flat regions, respectively (Artur et al. [26]). **l**–**r** Skyrmionic spin texture in CGT. **l**, **m** ${B}_{z}^{\text{lever}}(x,y)$ maps of the thick part of the sample at ${\mu }_{0}{H}_{z}=17$ mT (**l**) and $28$ mT (**m**), respectively, after field-cooling in 10 mT. **n**–**q** Corresponding $\frac{\partial {B}_{z}\left(x,y\right)}{\partial z}$ and magnetization ${M}_{z}(x,y)$ simulated at ${\mu }_{0}{H}_{z}=26$ (**n**, **p**) and $38$ mT (**o**, **q**), showing stripe domains shrink into bubbles at higher field. **r** The simulated magnetization configuration of the magnetic bubbles at ${\mu }_{0}{H}_{z}=38$, showing a Bloch-type skyrmionic texture in the middle layer, which gradually transforms into a Néel-type texture at the surface layers (Vervelaki et al. [25])

Magnetic bubbles and skyrmions are other notable nanoscale phenomena observed in CGT flakes, in addition to magnetic edges discussed above. Magnetic imaging techniques, including cryogenic Lorentz TEM (CLTEM) [26], MFM [20], SOL [25], and SOT, demonstrate the presence of bubble lattices with varying chirality in this material. Figure 10a–b shows two subsequent SOT images of magnetic islands with $w=1600$ nm, measured at ${\mu }_{0}{H}_{z}=- 90$ and $- 95$ mT, respectively (on the same area as in Fig. 5a). In addition to the well observed domains, smaller and weaker localized features are also seen, which can be attributed to magnetic bubbles. Subtracting the two images seen in Fig. 10a and b results in a magnetic field map difference, as depicted in Fig. 10c. In spite of the small difference in magnetic fields, the difference image (Fig. 10c) captures the evolution and deformation of these magnetic bubbles. Figure 10d illustrates the residual magnetic field obtained by subtracting two SOT images acquired at ${\mu }_{0}{H}_{z}=- 90$ mT and ${\mu }_{0}{H}_{z}=- 95$, showing a more distinct bubbles. Note that the type of skrymions and bubbles cannot be determined directly by SOT measurements since these are sensitive only to the OOP component of the magnetic field and not to the in-plane (IP) component.

McCray et al*.* used CLTEM to explore the formation of magnetic bubble lattices in CGT flakes [26]. The study reveals a range of magnetic textures, including homochiral and mixed-chirality bubble lattices, influenced by the topography of the CGT flakes. Figure 10h shows transport of intensity equation (TIE)—reconstructed images of the electron phase shift and integrated induction maps of the same region of a CGT sample after field-cooling it to 22 K in a 50-mT applied field. In Fig. 10e and g, the uniform positive phase shift across all bubbles indicates a homochiral lattice of right-handed chirality bubbles. After performing the same field-cooling procedure, a mixed-chirality bubble lattice was observed in the same region, as shown in Fig. 10f and h. Apart from the difference in chirality distribution, the two bubble lattices are similar, with the size and distribution of the bubbles being independent of the global lattice topology. The primary difference between the two lattices was the rare presence of topologically trivial type II bubbles that formed solely in mixed-chirality bubble lattices.

The formation of both mixed chirality and homochiral bubble lattices across multiple CGT samples was consistently observed. Whether a homochiral or mixed-chirality bubble lattice forms is a stochastic process: within any one region of a flake, repeated identical field-cooling runs could give rise to either a mixed-chirality or a homochiral lattice, which indicates that the effect is not due to microstructural or compositional variations. However, while flat regions of CGT sometimes contained homochiral bubble lattices, flake regions with topographical variations consistently showed mixed-chirality lattices, leading to a potential method for affecting the net topology of the magnetic domain structure in CGT.

Some wrinkles and folds in the flake can form during the exfoliation process. Figure 10i is of a region close to such a wrinkle and shows a phase image of a region that contains both homochiral and mixed-chirality lattices of magnetic bubbles. The region to the right of the image is flat, but the left side approaches a fold in the flake. The flat region contains predominantly homochiral bubbles, and an integrated induction map from this region is shown in Fig. 10j. Closer to the wrinkle on the left side of the image, bubbles of both chiralities were observed, similar to what is shown in Fig. 10f. However, unlike mixed-chirality bubble lattices in flat regions of the flake, here also deformed bubbles that are elongated into stripe domains are observed, as well as a higher concentration of topologically-trivial type-II bubbles, as shown in Fig. 10k.

Vervelaki et al. studied the thickness-dependent magnetic textures in few-layer CGT using scanning SOL [25]. To determine whether such skyrmionic bubbles are found in few-layer thick CGT, the sample was field-cooled at ${\mu }_{0}{H}_{z}=10$ mT. Figure 10l shows a measurement of ${B}_{z}^{\text{lever}}(x,y)$ over the thick part of the field-cooled sample after the applied field was increased to ${\mu }_{0}{H}_{z}=17$ mT. Stray field patterns characteristic of labyrinth domains are visible over most of the 15-layer thick region with some percolating features expanding to neighboring regions. As shown in Fig. 10m, after the field is further increased to ${\mu }_{0}{H}_{z}=28$ mT, the patterns related to labyrinth domains shrink and transform into bubble-like features. In corresponding micromagnetic simulations, the effect of field cooling procedure was studied, starting with an arbitrary labyrinth domain, applying ${\mu }_{0}{H}_{z}=10$ mT, and then letting the system relax to find the minimum energy state. The resulting simulated $\frac{d{B}_{z}\left(x,y\right)}{dz}$ and ${M}_{z}(x,y)$ at ${\mu }_{0}{H}_{z}=26$ mT presented in Fig. 10n and p, respectively, exhibit features similar to those measured in the field-cooled ${B}_{z}^{\text{lever}}(x,y)$. The corresponding ${M}_{z}(x,y)$ shows that stripe domains start forming at the edge of the flake, form multiple branches, and expand to the thinner regions. As the field is increased in the simulations to ${\mu }_{0}{H}_{z}=38 \text{mT}$, the corresponding $\frac{d{B}_{z}\left(x,y\right)}{dz}$ map in Fig. 10o qualitatively matches the bubble-like features in the measured ${B}_{z}^{\text{lever}}(x,y)$. ${M}_{z}(x,y)$ maps reveal that upon the increase of ${H}_{z}$, the underlying domains shrink and transform into bubbles, most of which have skyrmionic magnetization texture, as shown in Fig. 8q. The agreement between the SOL images and simulation, suggests that the spin texture behind the bubble-like features shown in Fig. 10m corresponds to skyrmionic bubbles. The skyrmionic bubbles also show a modulating helicity from the top to the bottom layer, as schematically shown in Fig. 10r. This behavior has been previously proposed in systems with skyrmions [43] and has also been observed for skyrmionic bubbles [44].

## Concluding Remarks

The edge magnetization in CGT is confined to a magnetic region with a width and thickness of tens of nanometers [22]. The microscopic physical mechanism responsible for edge magnetism has yet to be identified, but applying simple geometric considerations related to thickness variations along the edges can account for the experimental results quite well. A plausible explanation for the edge state is the presence of strain at the sample edges. Interestingly, a similar magnetic edge state observed in another material has been attributed to the Stoner mechanism [38]. However, applying this model to CGT is not straightforward, as CGT is insulating [9]. Further investigations are necessary to confirm the presence and nature of such states in CGT.

Beyond the intriguing fundamental science involved, edge magnetism holds great promise for spintronic applications. The non-magnetic nature of amorphous CGT combined with edge confinement introduces a method to control the local magnetic geometry, potentially influencing microscopic exchange interactions and enhancing ferromagnetic exchange over larger distances. Directly written magnetic structures could prove useful in devices requiring magnetic channels, offering significant potential for applications and fundamental research. Nanopatterning a 2-D vdW magnet into a square grid provides a versatile platform to explore a wide range of fundamental correlated phenomena. The dipolar coupling in such systems generates intricate interactions. Future studies could combine the effective antiferromagnetic interactions mediated by the dipolar field with lattice geometries that induce frustration, enabling the study of artificial spin-ice in magnetic 2-D materials. These findings open new pathways to connect spin-ice systems, frustration physics, and criticality with the ultrathin limits routinely achieved in 2-D materials.

The AHE has been reported in Pt/CGT [18] and Ta/CGT [17] devices, where the ferromagnetic CGT induces the AHE. The origin of AHE signal in heavy-metal/CGT heterostructures remains an area of ongoing research, where proximity effects from spin–orbit coupling or edge-induced magnetism could lead to practical applications and insights into 2-D materials.

Magnetic bubbles and skyrmions in CGT thin flakes exhibit unique lattice structures, forming homochiral or mixed-chirality configurations depending on topography and field-cooling conditions. Magnetic images and simulations reveal the evolution of labyrinth domains into skyrmionic bubbles with modulating helicity across layers [20, 21, 25, 26]. These findings highlight the stochastic nature of bubble formation and their potential applications in spintronics and frustrated magnetic systems.

In summary, this review focused on a small fraction of the vast nano-scale magnetic phenomena observed in the vdW ferromagnet CrGeTe_3_. These discoveries reveal the considerable promise of CGT and related 2-D magnetic materials for fundamental research and spintronic applications, underscoring the need for continued research into their rich nanoscale phenomena.

## Data Availability

No datasets were generated or analysed during the current study.
